# The Anti-Proliferative Effect of L-Carnosine Correlates with a Decreased Expression of Hypoxia Inducible Factor 1 alpha in Human Colon Cancer Cells

**DOI:** 10.1371/journal.pone.0096755

**Published:** 2014-05-07

**Authors:** Barbara Iovine, Giorgia Oliviero, Mariangela Garofalo, Maria Orefice, Francesca Nocella, Nicola Borbone, Vincenzo Piccialli, Roberto Centore, Massimiliano Mazzone, Gennaro Piccialli, Maria Assunta Bevilacqua

**Affiliations:** 1 Dipartimento di Medicina Molecolare e Biotecnologie Mediche, Università degli Studi di Napoli Federico II, Napoli, Italy; 2 Dipartimento di Farmacia, Università degli Studi di Napoli Federico II, Napoli, Italy; 3 Dipartimento di Scienze Chimiche, Università degli Studi di Napoli Federico II, Napoli, Italy; 4 Lab of Molecular Oncology and Angiogenesis, Vesalius Research Center, Leuven, Belgium; 5 Lab of Molecular Oncology and Angiogenesis, Vesalius Research Center, Department of Oncology, Leuven, Belgium; CNR, Italy

## Abstract

In recent years considerable attention has been given to the use of natural substances as anticancer drugs. The natural antioxidant dipeptide L-carnosine belongs to this class of molecules because it has been proved to have a significant anticancer activity both in vitro and in vivo. Previous studies have shown that L-carnosine inhibits the proliferation of human colorectal carcinoma cells by affecting the ATP and Reactive Oxygen Species (ROS) production. In the present study we identified the Hypoxia-Inducible Factor 1α (HIF-1α) as a possible target of L-carnosine in HCT-116 cell line. HIF-1α protein is over-expressed in multiple types of human cancer and is the major cause of resistance to drugs and radiation in solid tumours. Of particular interest are experimental data supporting the concept that generation of ROS provides a redox signal for HIF-1α induction, and it is known that some antioxidants are able to suppress tumorigenesis by inhibiting HIF-1α. In the current study we found that L-carnosine reduces the HIF-1α protein level affecting its stability and decreases the HIF-1 transcriptional activity. In addition, we demonstrated that L-carnosine is involved in ubiquitin-proteasome system promoting HIF-1α degradation. Finally, we compared the antioxidant activity of L-carnosine with that of two synthetic anti-oxidant bis-diaminotriazoles (namely **1** and **2**, respectively). Despite these three compounds have the same ability in reducing intracellular ROS, **1** and **2** are more potent scavengers and have no effect on HIF-1α expression and cancer cell proliferation. These findings suggest that an analysis of L-carnosine antioxidant pathway will clarify the mechanism underlying the anti-proliferative effects of this dipeptide on colon cancer cells. However, although the molecular mechanism by which L-carnosine down regulates or inhibits the HIF-1α activity has not been yet elucidated, this ability may be promising in treating hypoxia-related diseases.

## Introduction

L-Carnosine (β-Ala-His) is a naturally occurring histidine dipeptide, endogenously synthesized and widely found in the brain, muscle, kidney, stomach, and, in large amounts, in the skeletal muscle. This dipeptide has been proved to perform a number of biological functions, including anti-oxidant activity, ability to chelate metal ions, inhibition of protein glycosylation, anti-inflammatory and anti-senescence properties [1]. Another aspect of the effect of L-carnosine concerns its anti-proliferative effect in human cell lines. Recently, we have demonstrated that L-carnosine inhibits the proliferation of human colorectal carcinoma HCT-116 cells by affecting the ATP and ROS production and by inducing the cell cycle arrest in G1 phase [2]. In addition, some authors, with a proteomic approach, support the possibility that this dipeptide affects tumour cell growth in the human glioma cells and retards tumour growth in vivo in a NIH3T3-HER2/neu mouse model through an interference with protein folding/processing and HIF-1α signalling [3]–[4]. Actually, considerable efforts have been directed to the discovery of the chemical or natural molecules that target HIF-1α protein and regulate HIF-1α signalling pathway through a variety of molecular mechanisms, including transcriptional regulation, stabilization, degradation and transactivation. Of particular interest is the role of ROS and antioxidant molecules in HIF-1α regulation. Indeed, a series of compounds, such as rapamicin and resveratrol, have been shown to be inhibitors of HIF-1α [5]–[6]. HIF-1α is a component of HIF-1 complex that plays a central role in O_2_ homeostasis and, in fact, is considered a central regulator of the adaptation responses of cancer cells to hypoxia [7]. HIF-1 complex is a heterodimeric transcription factor consisting of O_2_-regulated HIF-1α and constitutively expressed HIF-1β subunits. Under normoxic conditions the isoform prolyl hydroxylase PHD2 hydroxylates HIF-1α on two functionally independent proline residues, Pro^402^ and Pro^564^, within the ODD (oxygen-dependent degradation) domain [8]–[9]. Hydroxylated Pro residues promote the recruitment of HIF-1α by Von Hippel-Lindau tumour suppressor protein (VHL), a recognition module of the E3-ubiquitin ligase, responsible for its ubiquitination and subsequent proteasome-mediated degradation [10]. Under hypoxic conditions the HIF-1α protein escapes to proteolysis, is upregulated, and forms a heterodimer with HIF-1β in the HIF-1 complex. The HIF-1 complex recognizes and binds to the hypoxia responsive element (HRE) of the hypoxia-inducible genes, including genes that influence angiogenesis, iron metabolism, modulation of glucose metabolism, cell proliferation, survival, and invasion, thereby activating their transcription [11]. In recent years, HIF-1 has emerged as a promising target for cancer therapeutics. In fact, HIF-1α over-expression is a common feature of human cancers, where it mediates the adaptation to the hypoxic tumour microenvironment. In accordance with these observations, the purpose of this study was to investigate in HCT-116 cell line the effects of L-carnosine on the expression of HIF-1α and HIF-1α-dependent genes. In addition, in recent years of particular interest it has been the role of ROS and antioxidant molecules in HIF-1α regulation [12]. Thus, to understand the mechanisms responsible for the L-carnosine effect we have also examined how this dipeptide affects ROS intracellular levels in comparison with two new anti-oxidant bis-diaminotriazole compounds (namely **1** and **2** respectively) available from our laboratories and whose antioxidant activity is unpublished. Despite these three compounds have the same ability in reducing intracellular ROS, we found that **1** and **2** have no effect on HIF-1α expression and cancer cell proliferation. We suppose that the antioxidant activity of L-carnosine operates with a different mechanism than compounds **1** and **2**. Thus, we conclude that an analysis of the L-carnosine antioxidant pathway will clarify the mechanism underlying the effects of this dipeptide on colon cancer cells.

## Materials and Methods

### Cell Culture

HCT-116 human colorectal carcinoma cell line was purchased from the American Type Culture Collection (ATCC) USA. The cell line was cultured at 37°C and 5% CO_2_ in DMEM (Dulbecco’s Modified Eagle Medium) purchased from BioWhittaker Classic Cell Culture Media, Lonza - VWR International s.r.l., supplemented with 10% fetal bovine serum (FBS) (Gibco Laboratories, North Andover, Massachusetts) and 1% penicillin/streptomycin (Gibco Laboratories).

### Chemical Synthesis of Bis-triazole Compounds 1 and 2

The two compounds were prepared by a reaction of the corresponding acid (trans-trans-muconic acid and fumaric acid, respectively) with diaminoguanidine monohydrochloride in polyphosphoric acid as the dehydrating agent, following the procedure already described in [13]. The chlorohydrates **1** and **2** were prepared as follows. Each bis-diaminotriazole was suspended in water. Diluted hydrochloric acid (10 mL conc. in 100 mL water) was added, heated to ebullition until the solid was completely dissolved. Upon cooling to room temperature, **2** was obtained as brown prismatic crystals. In the case of **1**, the chlorohydrate (pale brown prismatic crystals) was obtained upon addition of ethanol to the water solution and cooling to room temperature. The structure of the chlorohydrates and the protonation at the N-2 atom of the ring, instead of at the amino groups, was confirmed by single crystal analysis (work in preparation) and is consistent with similar results reported in [13].

### Treatments

L-carnosine, supplied by Sigma-Aldrich Canada, was dissolved in DMEM without phenol red (Sigma–Aldrich). HCT-116 cells were plated at a concentration of 3.0×10^6^ cells in 100 mm plates. After incubation for one day at 37°C in 5% CO_2_, L-carnosine was added from 0.5 to 100 mM; the cells were incubated at 37°C and harvested 24 h after. The treatment with the bis-diaminotriazole compounds (**1** and **2**) was carried out under the same conditions using concentrations from 0.05 to 0.5 mM. For CoCl_2_ treatment 100 mM CoCl_2_ was added to HCT-116 for 6 h. MG132, supplied by Calbiochem USA was dissolved in dimethyl sulfoxide (DMSO) and was added to the culture medium. HCT-116 cells were incubated for 1 h prior to L-carnosine treatment [14]. 24 h after L-carnosine treatment the cells were harvested for protein immunoprecipitation.

### Fluorescence Microscopy

HCT116 cells were grown on slides for 24 h and treated with 100 mM L-carnosine. After 24 h, cells were washed with PBS and fixed with cold 4% paraformaldehyde in PBS at room temperature for 10 min and then were permeabilized with 0.4% TRITON X100 in PBS 1X. After 10 min, cells were washed twice with PBS 1X/0.4% bovine serum albumin (BSA) for 1 h at room temperature. The cells were first treated with HIF-1α antibodies (Santa Cruz Biotechnology, Santa Cruz, CA) in PBS 1X/0.4% BSA for 1 h at room temperature and subsequently with ALEXA 568 (Molecular Probes’ Alexa Fluor 568) secondary antibodies in PBS 1X/0.4% BSA for 1 h at room temperature in the dark. After washing with PBS, cells were stained with mounting medium for fluorescence with DAPI (Vector laboratories). Results were examined by fluorescence microscopy on a Zeiss Axiophot at 40X resolution.

### Antioxidant Activity Measurement by the DPPH Method

DPPH (1,1-diphenyl-2-picrylhydrazyl) quenching method was employed for the evaluation of the antioxidant activity of **1**, **2** and L-carnosine [15]. The ability of the samples to scavenge the DPPH radical was measured using the Brand-Williams method [16]. Aliquots (60 µL) of 1 mM solution of **1**, **2** and L-carnosine were added to 3 mL of DPPH solution (6×10^−4 ^M) and the absorbance was determined at 515 nm (Philips PU 8620 series UV/Visible spectrophotometer) every 5 min until the steady state. For each antioxidant assay, a trolox aliquot was used to develop a 50–500 mM standard curve. All the data were then expressed as Trolox Equivalents (mmol TE/L).

### Analysis of Cell Viability

The MTT assay was performed according to the protocol previously reported [17]. HCT-116 cells were plated at a density of 1×10^5^ cells in 96-well plates. Subsequently, the bis-diaminotriazole compounds (**1** and **2**) were assayed using concentrations from 0.05 to 0.5 mM. After 24 h of incubation, 10 µL of 3-(4,5-dimethylthiazol-2-yl)-2,5-diphenyl tetrazolium bromide (MTT) (Sigma Aldrich) (0.05 mg/mL) were added to the culture medium. After 4 h at 37°C the culture medium was removed and 200 µL of DMSO were added to dissolve the salts of formazan. The absorbance was measured with a 96-wells plate spectrophotometer at 570 nm. The experiments were independently performed three times and each experiment contained triple replicates. Control samples containing a complete culture medium devoid of cells or control cells without L-carnosine were also included in each experiment.

### Clonogenic Assay

HCT116 cells were plated at a density of 500 cells/well in 12-well plates in 500 µL of fresh culture medium containing complete DMEM with 20–50–100 mM L-carnosine or bis-diaminotriazole compounds (**1** and **2**) (from 0.05 to 0.5 mM). After 7 days, the cells were washed twice with PBS 1X and stained with a solution of 0.2% crystal violet, 50% methanol and 10% acetic acid in H_2_O for 30 min at room temperature. Subsequently the cells were washed with deionized H_2_O and photographed.

### Measurement of Reactive Oxygen Species (ROS)

The formation of ROS was evaluated by means of the 2,7-dichlorofluorescein-diacetate probe (H2DCF-DA) (purchased from Sigma–Aldrich). HCT-116 cells were plated at a density of 5×10^3^ cells/well in 96-well plates in 100 µL of fresh culture medium containing DMEM without phenol red. Cells were allowed to grow for 24 h at 37°C in 5% CO_2_, then L-carnosine (from 0.5 to 100 mM) or compounds **1** and **2** (from 0.05 to 0.5 mM) were added to the culture medium. The ROS measure was performed according to the protocol previously reported [18]. Fluorescence was monitored using a LS 55 Fluorescence Spectrometer (Perkin–Elmer Ltd., Beaconsfield, England). In each experiment, the fluorescence increase was measured in three replicate cultures for each treatment.

### Transfection Assay and Luciferase Reporter Assay

The cells were transfected with the hypoxia response element (HRE)-luciferase reporter plasmid or with a vector containing the oxygen-dependent degradation (ODD) domain of HIF-1α, using Lipofectamine 2000 (Invitrogen, Darmstadt, Germany) in the serum-free OptiMEM-1 medium (Lonza, Verviers, Belgium), according to the manufacturer’s specifications. Cultures were maintained at 37°C in 5% CO_2_ for 24–48 h. Luciferase activity was evaluated by the Dual-Luciferase Reporter assay system (Promega, Madison WI, USA) and with a Promega GloMax 20/20 Luminometer, according to the manufacturer’s specifications.

### Preparation of Total, Nuclear and Cytosolic Protein Extracts

Total extracts of HCT-116 were prepared 24 h after treatment with L-carnosine from 0.5 to 100 mM or with **1** or **2** from 0.05 to 0.5 mM. The samples were washed twice with ice-cold PBS 1X and centrifuged at 240 g for 10 min at 4°C. The cell pellet was resuspended in a cold lysis buffer containing 1% Triton X100, 0.1% SDS, 0.1% NaN_3_, 100 mM NaCl, 50 mM NaF, 0.1 mM Na_3_VO_4_, 10 mM NaPPi, 0.5 mM PMSF and 10 µg/mL aprotinin, leupeptin and pepstatin at 4°C for 30 min on ice. The cell lysates were centrifuged at 20,000 g for 10 min at 4°C. For cytosolic and nuclear extracts HCT116 cells were harvested, washed twice with ice-cold PBS 1X and centrifuged at 240×g for 10 min at 4°C. The cell pellet was resuspended in 100 µL of ice-cold hypotonic lysis buffer (10 mM HEPES pH 7.9, 10 mM KCl, 1 mM PMSF, 0.1 mM EDTA, 0.1 mM EGTA, 1 mM DTT, 1 mM Na_3_VO_4_, 10 µg/mL each of aprotinin, leupeptin, and pepstatin) and incubated with shaking at 4°C for 15 min. Next, the cells were lysed by adding 5% NP40. The cellular extract was centrifuged for 5 min at 500×g and the supernatant containing the cytosolic fraction was then obtained after further centrifugation for 10 min at 20.000×g.

The nuclear pellet was resuspended in high salt extraction buffer (20 mM HEPES pH 7.9, 0.4 M NaCl, 1 mM PMSF, 1 mM EDTA, 1 mM EGTA, 1 mM DTT, 10 µg/mL each of aprotinin, leupeptin, and pepstatin) and incubated with shaking at 4°C for 15 min. The nuclear extract was then centrifuged for 15 min at 20.000×g, and the supernatant was aliquoted and stored at −80°C. Protein concentration was determined by a Bio-Rad protein assay kit (Bio-Rad Laboratories). For the western blot analyses, the proteins obtained were challenged with antibodies against HIF-1α (rabbit polyclonal antibody from Santa Cruz Biotechnology), aldolase A (ABNOVA), aldolase C (rabbit polyclonal antibody from Santa Cruz Biotechnology) and β-actin (mouse monoclonal from Santa Cruz Biotechnology). The signals were detected by using the ECL kit (GE Healthcare).

### Immunoprecipitation

HCT-116 cells were lysed in RIPA buffer (50 mM Tris-HCl pH 8.0, 250 mM NaCl, 0.1% SDS, 1% NP-40, 1% sodium deoxycholate, 5 mM EDTA, and 1 mM DTT) supplemented with protease inhibitors. The samples (800 µg) were precleared with 30 µL of Protein A/G PLUS-Agarose (Santa Cruz Biotechnology) for 2 h at 4°C. The anti-HIF-1α antibody (rabbit polyclonal antibody from Santa Cruz Biotechnology) was added to lysate and incubated overnight at 4°C and then 30 µL of Protein A/G PLUS-Agarose were added to the lysate. After 1 h at 4°C the proteins were recovered in Laemmli buffer, resolved on 8% denaturing gel and then subjected to immunoblotting.

### Real-time PCR Assay

Total RNA was prepared from HCT-116 cells by using the RNeasy mini kit (Qiagen) and was used to synthesize the cDNA with random hexanucleotide primers and MultiScribe reverse transcriptase (Invitrogen) at 48°C for 1 h. The cDNA was then amplified in an iCycler iQ real time PCR detection system (Bio-Rad Laboratories) using iQTM SYBR Green Supermix (Bio-Rad Laboratories). The Real-Time PCR reactions and relative quantification gene expression were performed as reported in [19]. The ratios between 2^–ΔΔ*CT*^ before treatment with L-carnosine and those calculated for the samples incubated with L-carnosine from 5 to 100 mM are expressed as fold changes.

### Statistical Analysis

Results were expressed as the mean ± SDs (standard deviations) of three independent experiments. The statistical analysis was performed using one-way analysis of variance ANOVA and P value for a multiple comparison test. The level of statistical significance was defined as **p<0.001, ***p<0.0001 [2]. The analyses were performed using GraphPad PRISM software (version 3.0; GraphPad Software, Inc.: La Jolla, CA, USA, 2002) on a Windows platform.

## Results

### L-carnosine Reduces HIF-1α Protein Levels Affecting its Stability in HCT-116 Cells

**Figure 1:**
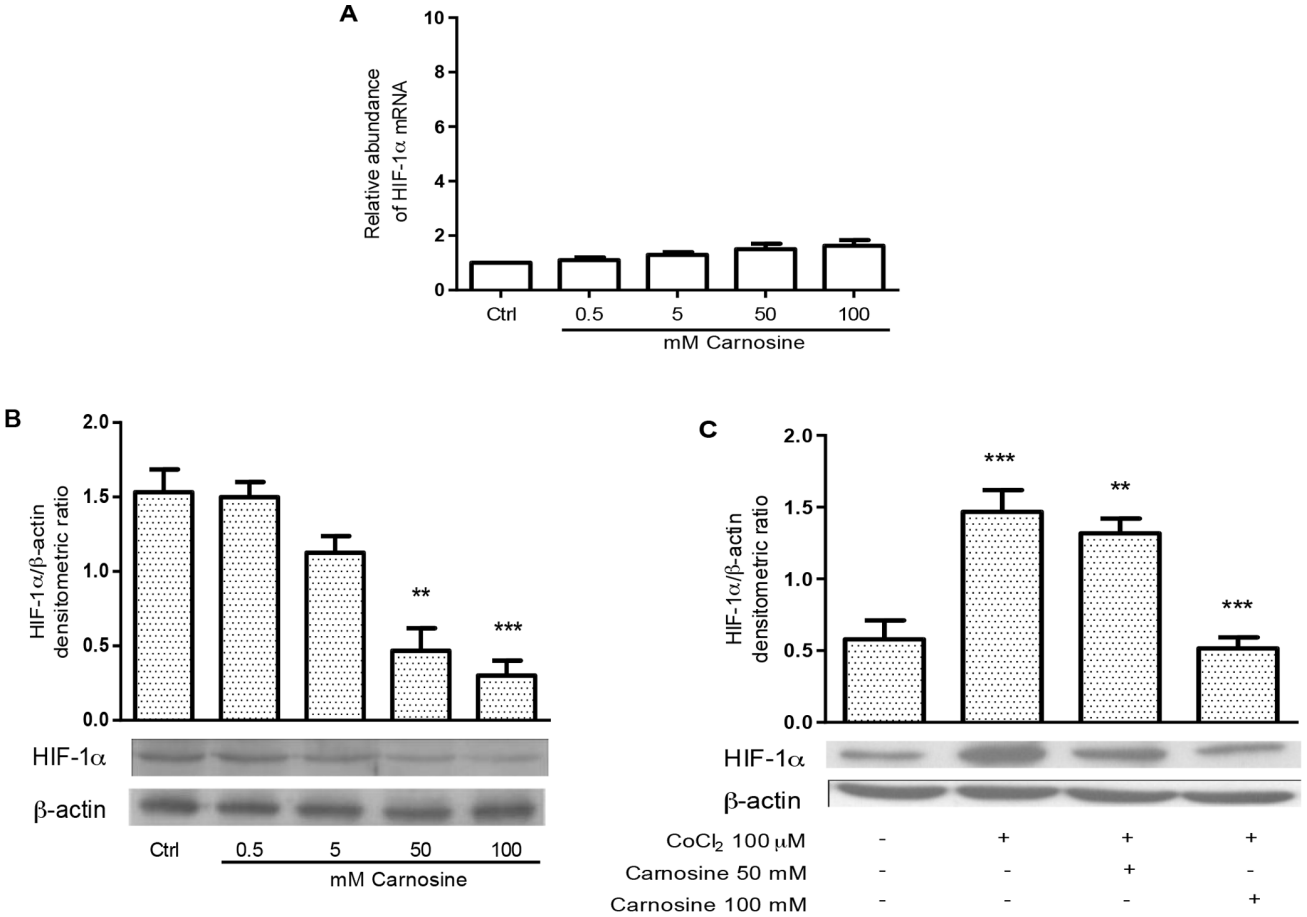
L-carnosine affects HIF-1α protein expression. (A) HIF-1α mRNAs were measured by real time PCR in a total RNA preparation from HCT-116 cells 24 h after L-carnosine treatment. White bars indicate fold changes of mRNA with respect to the amount of HIF-1α mRNA in untreated cells reported as value 1; (B) western blotting analysis of total protein extracts from HCT-116 cells 24 h after L-carnosine and (C) L-carnosine and CoCl_2_ treatment probed with HIF-1α and β-actin antibodies. Related histograms report the densitometric values of HIF-1α/β-actin ratio. The densitometric values represent the mean ± SDs of three independent experiments. The differences were considered significant at **p<0.001, ***p<0.0001.

**Figure 2:**
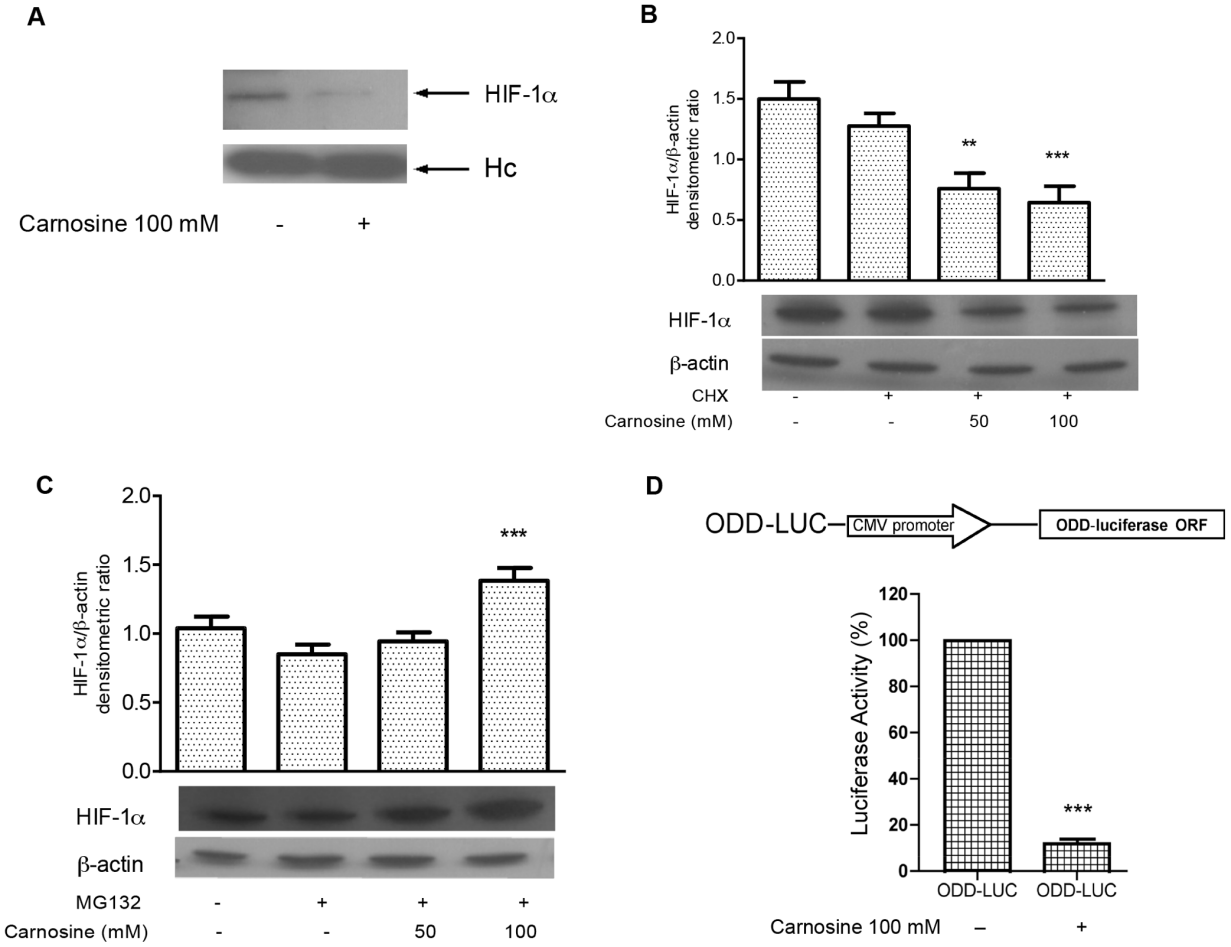
L-carnosine reduces the HIF-1α protein level affecting its stability and induces HIF-1α proteasome-dependent degradation. (A) Western blotting analysis probed with anti-HIF-1α antibody of immunoprecipitated proteins in HCT-116; (B) western blotting analysis of total protein extracts from HCT-116 cells 24 h after L-carnosine (100 mM) or L-carnosine (100 mM) plus 10 mM cycloheximide (CHX) treatment probed with HIF-1α and β-actin antibodies. Related histograms report the densitometric values of HIF-1α/β-actin ratio; (C) western blotting analysis of total protein extracts from HCT-116 cells 24 h after L-carnosine (100 mM) or L-carnosine (100 mM) plus 1 mM MG132 treatment probed with HIF-1α and β-actin antibodies. The densitometric values represent the mean ± SDs of three independent experiments. The differences were considered significant at **p<0.001, ***p<0.0001; (D) Schematic representation of ODD-LUC reporter vector. ODD-LUC vector contains the oxygen-dependent degradation (ODD) domain of HIF-1α cloned upstream from the luciferase gene. The luciferase activity was measured 48 h after transfection and represents the mean ± SDs of three independent experiments. The values have been reported as percentages of luciferase activity compared to 100% of untreated cells.

In order to establish the effect of the dipeptide L-carnosine on the expression of the HIF-1α transcription factor, human colon carcinoma cells (HCT-116) were treated with different concentrations of L-carnosine (from 0.5 to 100 mM). In particular, RT-PCR analysis, 24 h after L-carnosine addition, revealed a steady state of HIF-1α mRNAs levels, compared to untreated control cells (Figure 1A). Next, we evaluated the effects of L-carnosine on HIF-1α protein expression in the HCT-116 cells by western blot analysis. The protein levels were assessed both in hypoxia and normoxia, as well as in the presence (from 0.5 to 100 mM) and absence of L-carnosine. In the HCT-116 normoxic cells, 50–100 mM of L-carnosine reduced the expression of HIF-1α compared to a control sample not treated with L-carnosine (Figure 1B). We also observed that the hypoxia-induced (by CoCl_2_) expression of HIF-1α decreased after treatment with 50 or 100 mM L-carnosine (Figure 1C). The blots were re-probed with antibodies against β-actin to confirm the protein load equivalence. To confirm the reduction of HIF-1α we performed an immunoprecipitation assay. We found that the HIF-1α-antibody immunoprecipitate contained significant levels of HIF-1α protein only in the control sample (Figure 2A). In addition, we investigated the effect of L-carnosine upon HIF-1α protein synthesis in cells treated with cycloheximide (CHX). The analysis of HIF-1α protein stability in CHX-treated cells showed that the level of HIF-1α decreased in L-carnosine treated cells (50–100 mM), suggesting that L-carnosine affects the HIF-1α protein stability (Fig. 2B). Furthermore, we examined if this dipeptide could be involved in the proteasome degradation of HIF-1α. Therefore, we evaluated by western blot analysis the effect of the proteasome inhibitor MG132 on HIF-1α protein levels after treatment with 50–100 mM of L-carnosine. As shown in Figure 2C, the treatment with 1 mM MG132 and L-carnosine for 24 h increased the HIF-1α protein level compared to untreated control cells or to cells treated with MG132 alone. A further indication for the involvement of L-carnosine in HIF-1α proteasome degradation was obtained by transfection of a vector containing the ODD domain of HIF-1α. As shown in Figure 2D, the luciferase activity decreased noticeably 24 h after treatment with L-carnosine (100 mM).

### L-carnosine Influence HIF-1α Nuclear Translocation Reducing the Transcriptional Activity of Hypoxia Responsive Element (HRE) and the Expression of Its Downstream Genes

**Figure 3:**
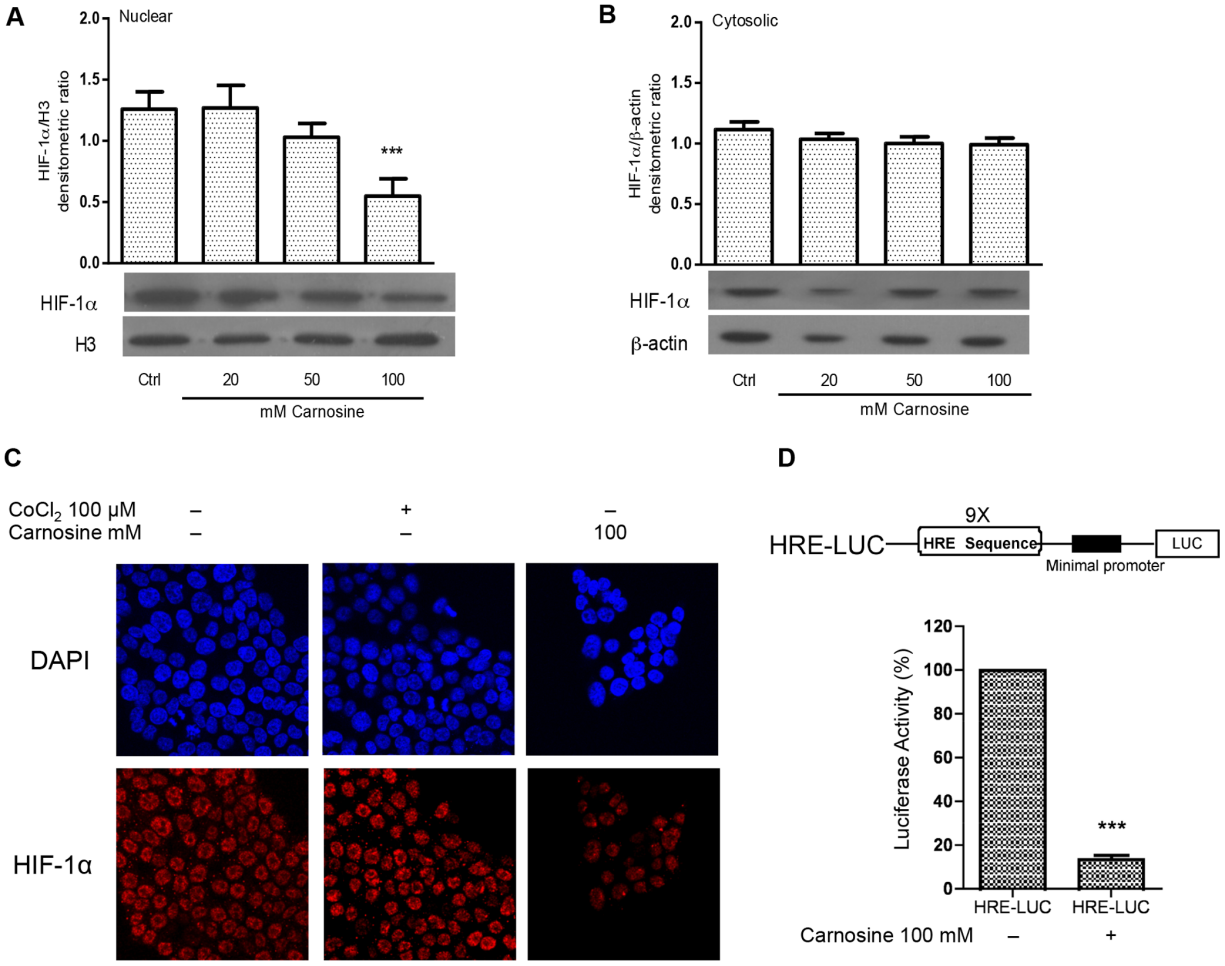
L-carnosine affects HIF-1α nuclear translocation reducing its transcriptional activity in HCT-116 cells. (A) Western blotting analysis of cytosolic and (B) nuclear protein extracts from HCT-116 cells 24 h after 20–50–100 mM L-carnosine treatment probed with HIF-1α, β-actin and Histone H3 antibodies. Related histograms report the densitometric values of HIF-1α/β-actin and HIF-1α/H3 ratio. The densitometric values represent the mean ± SDs of three independent experiments. The differences were considered significant at ***p<0.0001; (C) HCT-116 treated with CoCl_2_ and 100 mM L-carnosine for 24 h examined with fluorescence microscope at 40x magnification using filters for secondary antibody against HIF-1α protein and for 4′-6′-diaminodino-2-phenylindole (DAPI); (D) Schematic representation of HRE-LUC reporter vector. HRE-LUC vector contains nine repeat sequences of the Hypoxia Response Element (HRE) cloned upstream from the luciferase gene. The luciferase activity was measured 48 h after transfection and represents the mean ± SDs of three independent experiments. The values have been reported as percentages of luciferase activity compared to 100% of untreated cells.

**Figure 4:**
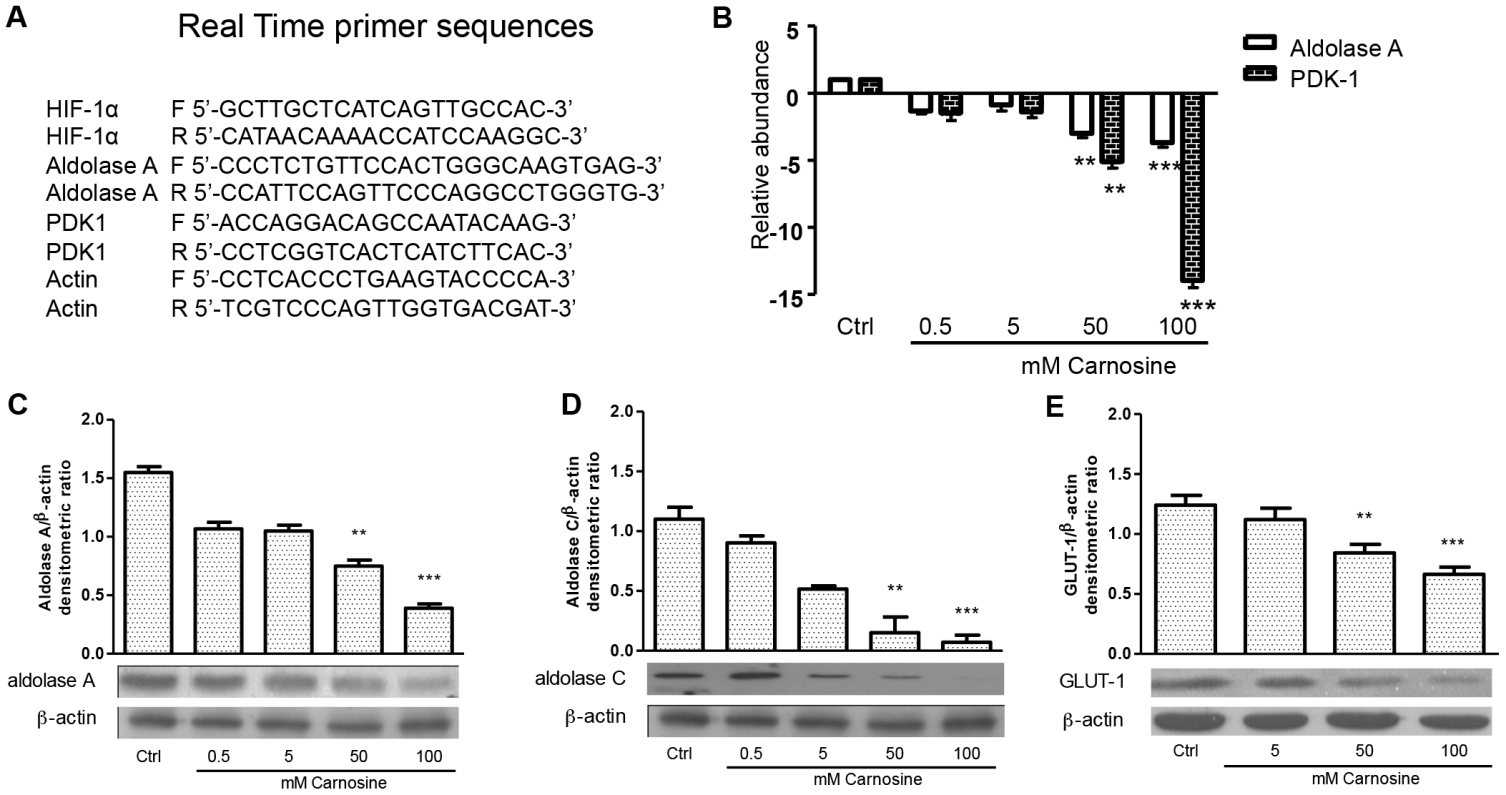
L-carnosine reduces HIF-1α dependent gene transcription in HCT-116 cells. (A) The table reports the primer sequences used in the Real Time PCR experiments; (B) aldolase A and PDK-1 mRNA_S_ were measured by real time PCR in a total RNA preparation from HCT-116 cells 24 h after L-carnosine treatment. Bars indicate fold changes of mRNA_S_ with respect to the amount of aldolase A and PDK1 mRNAs in untreated cells reported as value 1; (C), (D) and (E) western blotting analyses of total protein extracts from HCT-116 cells 24 h after L-carnosine treatment probed with aldolase A, Aldolase C, GLUT-1 and β-actin antibodies. Histograms report the densitometric values of aldolase A/β-actin ratio, aldolase C/β-actin ratio and GLUT-1/β-actin ratio. The densitometric values represent the mean ± SDs of three independent experiments. The differences were considered significant at **p<0.001, ***p<0.0001.

Stabilized HIF-1α protein translocates from the cytoplasm to the nucleus where it dimerizes with HIF-1β forming the transcriptionally active HIF-1 complex. Therefore, the nuclear localization of HIF-1α is essential for the transcriptional activity of HIF-1α. The effect of L-carnosine on the intracellular localization of HIF-1α was analysed at a first step by western blotting assay using cytoplasmic and nuclear protein fractions, and subsequently by immunofluorescence analysis. As shown in Figures 3A and 3B, L-carnosine influences the translocation of HIF-1α from the cytoplasm to the nucleus. In fact, when HCT-116 cells were treated with L-carnosine the nuclear protein levels of HIF-1α decreased noticeably, whereas the cytosolic levels were not affected. Also in the immunofluorescence analysis (Figure 3C) the HIF-1α signal in L-carnosine treated cells was weakly detectable compared to untreated control cells or to CoCl_2_-treated cells. In cancer cells, the activation of the HIF-1 complex entails its association with the HRE motif in the regulatory regions of target genes involved in the glycolytic process. For this reason, HCT-116 cells were transiently transfected with the HRE-luciferase reporter plasmid. As shown in Figure 3D, the treatment with 100 mM L-carnosine caused appreciable decrease in luciferase activity (24 h after the treatment). Subsequently, we evaluated the mRNAs of some HIF-1α-dependent genes. We measured by RT-PCR analysis the effects of different concentrations of L-carnosine (0.5, 5, 50 and 100 mM) on mRNA levels of aldolase A and PDK-1. As shown in Figure 4B, aldolase A and PDK-1 mRNAs levels decreased after treatment with L-carnosine, especially between 50 and 100 mM. The western blot analysis of aldolase A demonstrated also a reduction of the protein level (Figure 4C). In addition, we observed that the expression of the GLUT-1 protein, as well as of the aldolase C protein, decreased after L-carnosine treatment (Figure 4D and 4E). Aldolase C is a brain-specific isoform of aldolase, but recently it has been demonstrated that the mRNA level of aldolase C is 30-fold higher in human gastric cancer cell lines. The oligonucleotide sequences used in the RT-PCR analyses are reported in Figure 4A.

### The Antioxidant Effect of Compounds 1 and 2 Compared to L-carnosine

**Figure 5:**
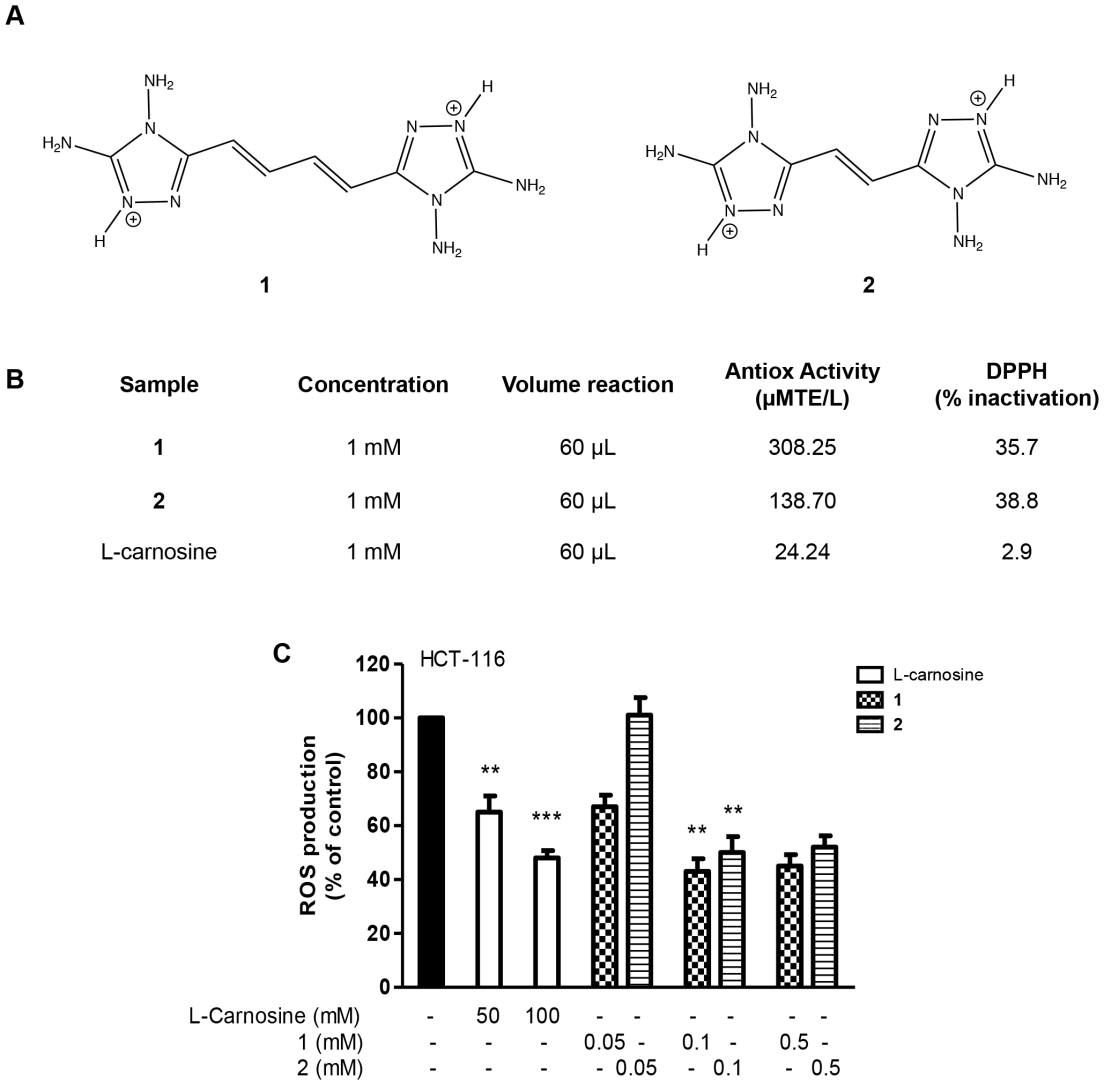
Compounds 1, 2 and L-carnosine strongly reduce the production of intracellular ROS. (A) Chemical structures of compounds **1** and **2**; (B) Antioxidant activity of L-carnosine, **1** and **2** measured by the DPPH method as described in Section 1.2.4; (C) ROS production evaluated 24 h after the addition of L-carnosine, **1** and **2** in HCT-116 cells by the probe 2′,7′-dichlorofluorescein-diacetate (H2DCF-DA). All values represent the mean ± SDs of three independent experiments and are expressed as a percentage compared to 100% of control cells. The differences were considered significant at **p<0.001, ***p<0.0001.

**Figure 6:**
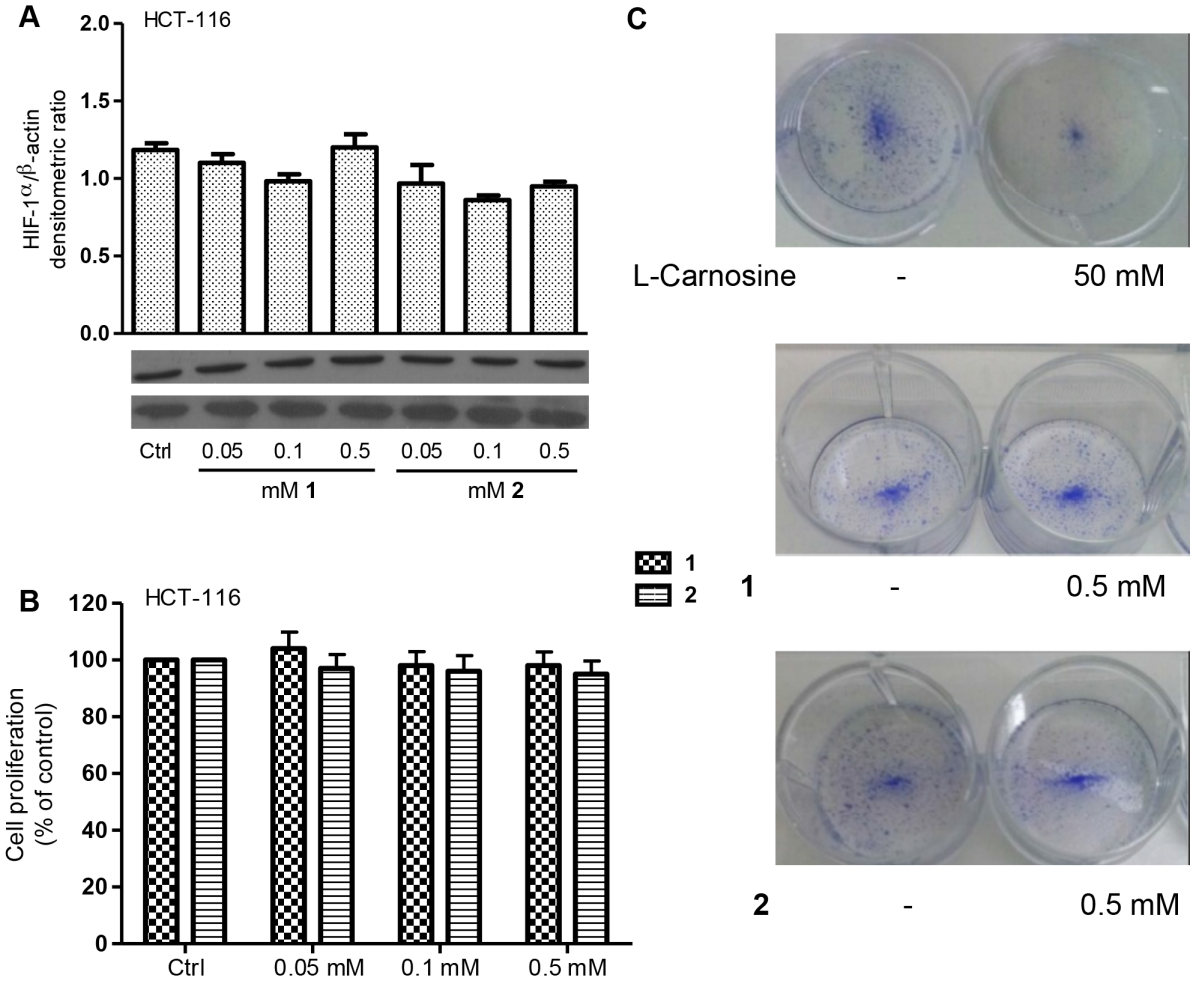
Compounds 1 and 2 have no effect on the HIF-1α protein level and cell proliferation in HCT-116. (A) Western blotting analysis of the total protein extracts from HCT-116 cells after treatment with compounds **1** and **2** probed with HIF-1α and β-actin antibodies. Histograms report the densitometric values of HIF-1α/β-actin ratio. The densitometric values represent the mean ± SDs of three independent experiments; (B) the cell proliferation was measured by MTT assay in HCT-116 cells 24 h after treatment with compounds **1** and **2**. All values represent the mean ± SDs of three independent experiments and are expressed as a percentage compared to 100% of control cells; (C) Clonogenic survival assay in HCT-116 after treatment with L-carnosine (50 mM) or compounds **1** and **2** (0.5 mM) with respect to untreated cells.

The generation of Reactive Oxygen Species (ROS) during hypoxia provides a redox signal for HIF-1α induction. In fact, some authors define ROS as positive regulators of HIF-1α [20]. The observation that other antioxidant substances can influence the level of HIF-1α protein led us to evaluate the antioxidant activity of two novel bis-diaminotriazole compounds (**1** and **2**) (Figure 5A), in comparison to that of L-carnosine, by DPPH assay. First, we measured the percentage of DPPH inactivation using a 1 mM concentration for all compounds, and we demonstrated that compounds **1** and **2** possess a major activity as ROS scavengers compared to L-carnosine (see the Table in Figure 5B). Next, we examined the intracellular ROS generation induced by L-carnosine (100 mM) in HCT-116 cells and compared it with the amount of ROS generated by treating the same cell line with different concentrations (from 0.05 to 0.5 mM) of compounds **1** and **2**. As shown in Figure 5C, the treatment with, 0.1 and 0.5 mM of **1** and **2** reduced the intracellular ROS generation in HCT-116 by about 40–50%, similarly to 50–100 mM L-carnosine. Finally, we also evaluated the effect of different concentrations of **1** and **2** (from 0.05 to 0.5 mM) on the HIF1-α expression and cell viability. As shown in Figures 6A and 6B, the addition of **1** and **2** did not affect the HIF-1α protein levels nor the cell proliferation. In addition, we evaluated by clonogenic survival assay the ability of HCT-116 cells to proliferate and to form a large colony or a clone in the presence of L-carnosine or **1** or **2** (Figure 6C). 50 mM L-carnosine reduced significantly the number of colonies with respect to the untreated control sample, whereas compounds **1** and **2** did not influence the cells ability to form colonies at the tested concentration. A flow cytometry assay (FACS) confirmed that **1** and **2** did not affect the cell cycle and did not induce apoptosis in HCT-116 cells (data not shown). The early apoptotic death rate in HCT-116 cells treated with the two compounds was not significantly different from the control sample.

## Discussion

In this study, we evaluated the effect of L-carnosine treatment on HIF-1α activity in human colon cancer cells. It has been reported that HIF-1α protein is over-expressed in many types of human cancer, including lung, breast, prostate, stomach, and colon carcinomas [21]–[22]. The expression of HIF-1 is enhanced by genetic alterations in tumour suppressor genes (p53, PTEN) and oncogenes (v-src, HRAS) and by the induction of several growth factors (IGF1 and IGF2, the basic fibroblast growth factor, and the EGF) [23]–[24]. Indeed, the homogeneous HIF-1 immunostaining pattern in several colon carcinoma cell lines is associated with these factors [25]. Particularly, in HCT-116 colon carcinoma cells, HIF-1α and HIF-2β regulate the cancer metabolism by overlapping with the KRAS oncogene [26]. We previously demonstrated that L-carnosine could abate ROS induced by the KRAS oncogene as well as the production of ATP [2]. In addition, an investigation with a proteomic approach supports the possibility that the dipeptide L-carnosine affects tumour cell growth in the human glioma cells by interfering with protein folding/processing or with HIF-1α signalling [4]. According to these observations, we have found, in HCT-116 cell line, that the levels of HIF-1α mRNAs are steady in the presence of L-carnosine, thus indicating that HIF-1α is not regulated at the transcription level. This result is not surprising because the expression of HIF-1α mRNA seems to be constitutive [27]. Nevertheless, in the same conditions this dipeptide decreased HIF-1α protein level, acting on its stability. Indeed, we have treated HCT-116 cells with L-carnosine in presence of cycloheximide, to inhibit the de novo protein synthesis, or in the presence of the proteasome inhibitor MG132. We found that L-carnosine reduced HIF-1α stability and induced its proteasome degradation. In addition, the transfection assay with the ODD domain-vector suggests that L-carnosine somehow acts via the oxygen-dependent degradation domain. Another interesting point concerns the finding that L-carnosine treatment affected the nuclear amount of HIF-1α, because the nucleus localization of this transcriptional factor is required to its transcriptional activity. For this reason, HCT-116 cells were transiently transfected with the hypoxia response element (HRE)-luciferase reporter plasmid, containing the sequence recognized by HIF-1α. We have found that in the cells treated with L-carnosine the luciferase activity decreased appreciably compared to the untreated control cells. Since HIF-1α represents one of the main responsible for the metabolic switch from mitochondrial respiration to glycolysis (Warburg effect) in tumour cells, we analysed some HIF-1α -dependent genes, involved in these molecular pathways. Then we evaluated the expression of Aldolase A and PDK-1 by Real Time PCR. The mRNAs levels of the aldolase A and PDK-1 decreased as well as GLUT-1, aldolase A and C protein levels. In particular, aldolase C is a brain-specific isoform of aldolase and it has been demonstrated that the mRNA level of this enzyme is 30-fold higher in human gastric cancer cell lines. [28]. In brief, L-carnosine acts on HIF-1α signalling decreasing its protein level and consequently its transcriptional activity. In recent years, it has been of particular interest the role of ROS and of antioxidant molecules in HIF-1α regulation [12]. Indeed, several experimental data demonstrate the ability of some antioxidants to suppress tumorigenesis by inhibiting HIF-1α [29]. Therefore, we have evaluated the effect on HIF-1α expression of two compounds, **1** and **2** in Figure 6A, obtained by organic synthesis and prepared by the reaction of the corresponding trans-trans-muconic and fumaric acid, respectively. We found that **1** and **2** did not influence the cell viability and HIF-1α protein level, although they have the same ability in reducing intracellular ROS levels compared to L-carnosine in HCT-116 cell line. We have also demonstrated by DPPH assay that these compounds, unlike L-carnosine, have a good activity as ROS scavengers. This finding could be not surprising because it has been reported that L-carnosine could exercise its antioxidant activity inhibiting the radical production by binding copper ions, interacting with lipid peroxidation products and inhibiting AGE (Advanced Glycation End product) formation, which are end products of the non-enzymatic glycation of proteins. Indeed, AGEs bind the receptors for AGEs (RAGE) on the cellular membrane, inducing oxidative stress and increasing ROS production [30]. It has also been demonstrated that L-carnosine prevents AGE formation in vivo and reacts with protein carbonyl groups protecting the macromolecules against their cross-linking actions [31]. In addition, Bento et al. propose a model for methylglyoxal-dependent degradation of HIF-1α, in which MGO promotes an increased association of Hsp40/70. This association leads to the recruitment of the carboxyl terminus Hsc70-interacting protein (CHIP) and, consequently, the polyubiquitination and proteasome degradation of HIF-1α [32]. However, a proteomics study in glioblastoma cells treated with L-carnosine shows a decrease in the protein expression of BAG2, involved in the inhibition of the ubiquitin ligase activity of CHIP. Therefore, the reduction of this protein may destabilize HIF-1α [4]. In accordance with these observations we presume that the analysis of the mechanism by which L-carnosine reduces ROS level and mostly the study of its potential targets could be critical to elucidate how L-carnosine influences HIF-1α as well the mechanism underlying its anti-proliferative effect on colon cancer cells. Further studies will also be needed to assess the possible involvement of PHDs in the HIF-1α degradation and to evaluate the effect on HIF-1α of natural inhibitors of nitric oxide (NO), available from our laboratories, since it has been demonstrated that NO can regulate HIF-1α activation [33]–[34].
