# Correction: The Anti-Proliferative Effect of L-Carnosine Correlates with a Decreased Expression of Hypoxia Inducible Factor 1 alpha in Human Colon Cancer Cells

**DOI:** 10.1371/journal.pone.0099739

**Published:** 2014-06-04

**Authors:** The PLOS ONE Staff

The Data Availability statement does not appear on the paper. The correct Data Availability statement is: The authors confirm that all data underlying the findings are freely available without restriction. All data are included within the paper.

The publisher apologizes for this error.
