# Correction: The Anti-Proliferative Effect of L-Carnosine Correlates with a Decreased Expression of Hypoxia Inducible Factor 1 alpha in Human Colon Cancer Cells

**DOI:** 10.1371/journal.pone.0105664

**Published:** 2014-08-11

**Authors:** The PLOS ONE Staff

There is an error in affiliations 4 and 5 for the author Massimiliano Mazzone. Affiliation 4 should be:

Lab of Molecular Oncology and Angiogenesis, Vesalius Research Center, VIB, Leuven, Belgium

Affiliation 5 should be:

Lab of Molecular Oncology and Angiogenesis, Vesalius Research Center, Department of Oncology, K.U. Leuven, Leuven, Belgium
